# Dublin Castle and the Royal College of Surgeons

**Published:** 1921-04-09

**Authors:** 


### Dublin Castle and the Royal College of Surgeons.

### THE MILITARY ORDER TO HOSPITALS.

When we recorded last week the new duty imposed upon the secretaries of Irish hospitals under the Restoration of Order in Ireland Act "to inform the military authorities daily of the names and particulars of persons treated for gunshot and similar wounds" we restrained criticism, because the nature of the particulars required was not known in detail.

The Order, apart from unofficial grumbling, has now evoked a protest from the Royal College of Surgeons in Ireland. In reply to representations the Council of the College has passed the following resolution:—

The Council are of opinion that it is contrary to the public interest that medical men should break their professional tradition, and without the consent of their patients disclose information which they have obtained in the discharge of their professional duties.

If any instance is reported to the Council in which one of the Fellows or Licentiates of this College is pressed to break confidence, such specific case will be considered by the Council, and representations made to the authorities, if such be thought necessary.

This resolution has evoked the following official statement from Dublin Castle:—

In answer to a question as to the meaning of the order recently issued to hospitals requesting them to furnish details of wounded patients admitted, it was officially stated that the smuggling into public hospitals and subsequent medical treatment there in secret of Republican gunmen injured in street attacks on Crown forces has been a considered policy on the part of Sinn Fein.

This is clearly shown in documents recently captured. They indicate that where practicable wounded Republicans are surgically treated in private at the expense of the I.R.A. funds by selected medical men, who have not made public any suspicions they may have had as to how the wounds were caused.

### Hospitals Charged with Concealing Facts.

But in serious cases wounded Republicans have been got into public hospitals often under assumed names, and there is ground for stating that in many cases both their presence and the nature of their wounds have been wilfully concealed.

It was with a view to checking this use of public institutions for the hiding and harbouring of evil-doers that the military order was issued requesting secretaries and doctors of hospitals to furnish daily particulars of the wounded admitted for treatment.

The recent protest against this order by the Council of the Royal College of Surgeons, Ireland, who contend that it entails a disclosure of information obtained in the discharge of their professional duties, is the more surprising in view of the fact that the details sought by the authorities are no more than those which are entered in the porter's admission book of any well-regulated hospital.

We should be glad to publish the official form which the military doubtless submit to hospital secretaries, for this would settle the nature of the particulars. Whatever these prove to be, the hospitals, as we pointed out last week, are inclined to resent a new duty so quickly imposed after the fate which met their recent demand for a more adequate grant.

